# Dissociable early attentional control mechanisms underlying cognitive and affective conflicts

**DOI:** 10.1038/srep37633

**Published:** 2016-11-28

**Authors:** Taolin Chen, Keith M. Kendrick, Chunliang Feng, Shiyue Sun, Xun Yang, Xiaogang Wang, Wenbo Luo, Suyong Yang, Xiaoqi Huang, Pedro A. Valdés-Sosa, Qiyong Gong, Jin Fan, Yue-Jia Luo

**Affiliations:** 1 Huaxi MR Research Center (HMRRC), Department of Radiology, West China Hospital of Sichuan University, Chengdu 610041, PR China; 2 State Key Laboratory of Cognitive Neuroscience and Learning, Beijing Normal University, Beijing, 100875, PR China; 3 Key Laboratory for Neuroinformation, Center for Information in Medicine, School of Life Science and Technology, University of Electronic Science and Technology of China, Chengdu 611731, PR China; 4 Department of Psychology, Beijing Forestry University, Beijing 100083, PR China; 5 School of Sociology and Psychology, Southwest University for Nationalities, Chengdu, China; 6 Department of Psychology, Queens College, City University of New York, New York, USA; 7 Shenzhen University, Shenzhen 518060, PR China; 8 Shenzhen Institute of Neuroscience, Shenzhen 518057, PR China; 9 Collaborative Innovation Center of Sichuan for Elder Care and Health, Chengdu Medical College, Chengdu, 610500 PR China

## Abstract

It has been well documented that cognitive conflict is sensitive to the relative proportion of congruent and incongruent trials. However, few studies have examined whether affective conflict processing is modulated as a function of proportion congruency (PC). To address this question we recorded event-related potentials (ERP) while subjects performed both cognitive and affective face-word Stroop tasks. By varying the proportion of congruent and incongruent trials in each block, we examined the extent to which PC impacts both cognitive and affective conflict control at different temporal stages. Results showed that in the cognitive task an anteriorly localized early N2 component occurred predominantly in the low proportion congruency context, whereas in the affective task it was found to occur in the high proportion congruency one. The N2 effects across the two tasks were localized to the dorsolateral prefrontal cortex, where responses were increased in the cognitive task but decreased in the affective one. Furthermore, high proportions of congruent items produced both larger amplitude of a posteriorly localized sustained potential component and a larger behavioral Stroop effect in cognitive and affective tasks. Our findings suggest that cognitive and affective conflicts engage early dissociable attentional control mechanisms and a later common conflict response system.

Attentional control refers to the ability to select and maintain actions in accordance with ultimate goals by ignoring task-irrelevant information^1^, and is typically probed in tasks wherein different incompatible response tendencies are simultaneously induced^2^,^3^. For instance, participants in the traditional Stroop task are asked to name the color of the printed words that possess congruent (e.g., RED printed in red) or incongruent (RED printed in green) semantic meanings. Incongruent stimuli consistently induce increased response times (RT) and error rates relative to congruent stimuli (i.e., interference effects), reflecting enhanced competition for attentional resources in response to incongruent compared to congruent stimuli^4^. In other words, more attentional control (i.e., attentional selection or adjustment) is needed for appropriate responses to incongruent stimuli. The attentional control mechanism underling cognitive conflict is modulated by the relative proportion of congruent and incongruent trials, with high proportions of congruent trials leading to large interference effects, i.e., a proportion congruency (PC) effect^5^,^6^. The PC effect may reflect a high-level strategic adjustment, manifested as a proactive top-down attentional control in the low proportion congruency (LPC) context and a reduced engagement of reactive control in the high proportion congruency (HPC) context^7^,^8^.

The PC effect on cognitive conflict can be detected by scalp event-related potentials (ERP) brain recording methods which allow a high temporal resolution. Previous findings have identified an early conflict-related component (i.e., N2) that exhibits a similar PC effect as at the behavioral level. In particular, the amplitude of the conflict-related N2 is augmented in the LPC context^9^,^10^ (but see also ref. 11), and this is associated with reduced interference effects. These effects echo the conflict-monitoring theory which maintains that infrequent incongruent trials in the HPC context decrease the level of control, producing a stronger interference effect; in contrast, frequent incongruent trials in the LPC context presumably lead to a steady maintenance of a high level of control, producing a weaker interference effect^12^. Furthermore, the sustained potential (SP), a late conflict-related component, is also modulated by the PC, such that its amplitude is augmented and associated with a larger interference effect in the HPC context compared to the LPC one^9^,^11^,^13^,^14^. Regarding the localization of these conflict-relevant components, accumulating evidence indicates that the N2 component is generated in the dorsal anterior cingulate (dACC)^15^,^16^ or the dorsolateral prefrontal cortex (DLPFC)^17^,^18^, reflecting the detection of conflict or conflict maintenance, while the SP component is generated in the lateral frontal and extrastriate cortices and thought to reflect conflict resolution^4^,^19^.

Affective conflict constitutes another important type of conflict that involves emotional stimuli and may engage different mechanisms of attentional control to those of cognitive conflict^20^,^21^. In view of the importance of affective conflict in the context of emotion regulation and affective disorders^22^,^23^,^24^, the past decade has witnessed an increasing interest in studying the psychological and neural signatures underlying its resolution^25^,^26^,^27^,^28^. Early studies on affective conflict often assessed the influence of emotional distractors on individual performance on target stimuli, i.e., naming the color of aversive words (e.g., “disgust” printed in red)^29^. However, emotional distractors and target stimuli employed in these tasks are neither semantically incongruent nor do they induce incompatible response tendencies. Accordingly, these tasks are unable to induce affective conflict that is comparable to cognitive conflict induced by the classical Stroop task^25^,^30^. Ektin *et al*. (2006) have developed a new word-face paradigm that allows for direct comparisons to be made between affective and cognitive conflicts. Specifically, participants are presented with facial expressions (e.g., fearful or happy) overlaid with congruent or incongruent emotional labels (e.g., “fearful” or “happy”), and asked to judge the facial expressions while ignoring the distractor of emotional word labels across the face^25^. As such, the affective conflict is derived from incompatible response tendencies between emotional expressions and word labels^8^,^25^. To compare the affective and cognitive conflicts, participants often perform another comparable cognitive conflict task, wherein they are asked to judge the gender of the faces while ignoring the distractor of gender word labels across the faces^26^,^31^,^32^.

Recent functional magnetic resonance imaging (fMRI) studies have compared the PC effect on conflict-related brain activations in affective and cognitive word-face Stroop tasks^31^,^33^, and identified similar actions on the dACC but different ones on the dorsal striatum and anterior insula^33^, suggesting both overlapping and distinct attentional control mechanisms underlying cognitive and affective tasks. Utilizing the high temporal resolution afforded by the ERP technique, we recently compared the modulation of PC on cognitive and affective conflict processing at distinct temporal stages in a flanker task using word stimuli^34^. The PC effect on a central N450, as an extension of the early central N2, was enhanced in the LPC context during cognitive tasks, whereas it was reduced during affective tasks. This differential PC effect on the N450 was localized in the DLPFC, with activity being increased in the cognitive task and reduced in the affective one. Furthermore equivalent PC effects were found on a parietal SP component during cognitive and affective tasks^34^. These findings generally echo observations of a recent study comparing cognitive and affective interference effects^35^. In particular, the authors identified greater N2 amplitude in the affective than the cognitive task regardless of stimulus congruency. In addition, they observed a stronger interference effect on N450 amplitude in the cognitive than the affective task, whereas the interference effect on the SP component was comparable in both tasks^34^. Taken together, previous ERP findings indicate that cognitive and affective conflict processing engage an early dissociable attentional control mechanism but a later common conflict response system.

Building on previous findings, here we further compared the PC effects on cognitive and affective conflicts with cognitive and affective versions of a commonly-used face-word Stroop task^26^,^31^,^33^,^36^, where the relative proportion of congruent and incongruent trials were varied in each block. We postulated that comparisons between the impact of cognitive and affective conflicts would allow us to assess the extent to which current models of attentional control based primarily on evidence on cognitive conflict effects would also be applicable to affective conflict. Although our previous ERP study has shed light on the modulation of the temporal dynamics of both cognitive and affective conflict processing by PC, it remains unclear whether the effects were specific to the revised flanker task using word stimuli, given that complex semantic processing may influence the time course of the conflict-related ERP components^37^,^38^. This potential confound was addressed in the current study by employing faces as stimuli^26^,^31^,^32^. In line with previous findings^34^, we hypothesized the early conflict-sensitive N2 component originating in the DLPFC would be augmented in the LPC context during the cognitive conflict task whereas the opposite would occur during the affective conflict task. We further hypothesized equivalent PC effects on the later parietal SP component during the cognitive and affective tasks.

## Results

### Behavioral performance

**Figure 1:**
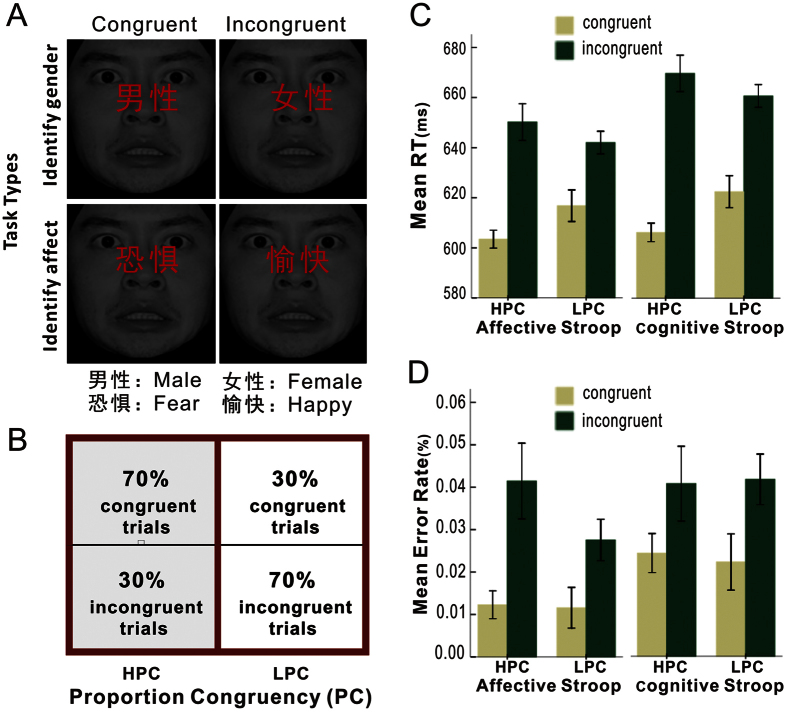
Experimental protocol and behavioral results. (**A**) The experimental design regarding Task types (cognitive task, affective task) and Stimulus congruence (congruence, incongruence). (**B**) Left panels: the high proportion congruency (HPC) block including 70% congruent trials and 30% incongruent trials; Right panels: the low proportion congruency (LPC) block including 30% congruent trials and 70% incongruent trials. (**C**,**D**) Left panels: mean RTs and mean error rates for congruent (yellow) and incongruent (green) trials in the HPC and the LPC contexts during affective task. Right panels: mean RTs and mean error rates for congruent (yellow) and incongruent (green) trials in the HPC and the LPC contexts during the cognitive task. The error bars represents one standard error.

A 2 (Task: cognitive, affective) × 2 (Proportion congruent: high, low) × 2 (Congruency: congruent, incongruent) repeated measures ANOVA was conducted on the RTs (Fig. 1C) and error rates (Fig. 1D). There was no main effect of Task for either RTs or error rates, suggesting that the two tasks were comparable in difficulty. A main effect of Congruency was identified for both RTs (F_1, 21_ = 100.81, *p* *<* 0.001, *η*^2^ = 0.83) and error rates (F_1, 21_ = 15.09, *p* *<* 0.005, *η*^2^ = 0.42), revealing slower responses (657 ms vs. 613 ms) and more error rates (0.04 vs. 0.02) in response to incongruent than to congruent trials (i.e., the interference effect). Furthermore, significant interactions of Congruency × Task (F_1, 21_ = 5.12, *p* *<* 0.05, *η*^2^ = 0.20) and Congruency × Proportion congruent (F_1, 21_ = 28.33, *p* *<* 0.001, *η*^2^ = 0.57) were observed for RT. Follow-up analyses showed that the interference effect was found in both cognitive (F_1, 21_ = 80.44, *p* *<* 0.001, Congruent vs. Incongruent: 616 ms vs. 666 ms) and affective (F_1, 21_ = 57.39, *p* *<* 0.001, Congruent vs. Incongruent: 610 ms vs. 647 ms) tasks, with no significant difference between the magnitude of this interference effect in the two tasks (*p* > 0.05). The interference effect was also found in both HPC (F_1, 21_ = 103.23, *p* < 0.001, Congruent vs. Incongruent: 606 ms vs. 661 ms) and LPC contexts (F_1, 21_ = 56.11, *p* < 0.001, Congruent vs. Incongruent: 620 ms vs. 652 ms) in the two tasks. In addition, a significantly larger interference effect was induced in the HPC context than the LPC one (*t*_1, 21_ = 3.45, *p* < 0.001, 55 ms vs. 32 ms). No other significant main effects or interactions were found (*p* > 0.05) for RTs or error rates. These results showed that both the cognitive and affective tasks had robust interference effects which were modulated by the PC, as indexed by attenuated interference effects in the LPC context compared with the HPC one.

### ERP Results

#### N1

**Figure 2:**
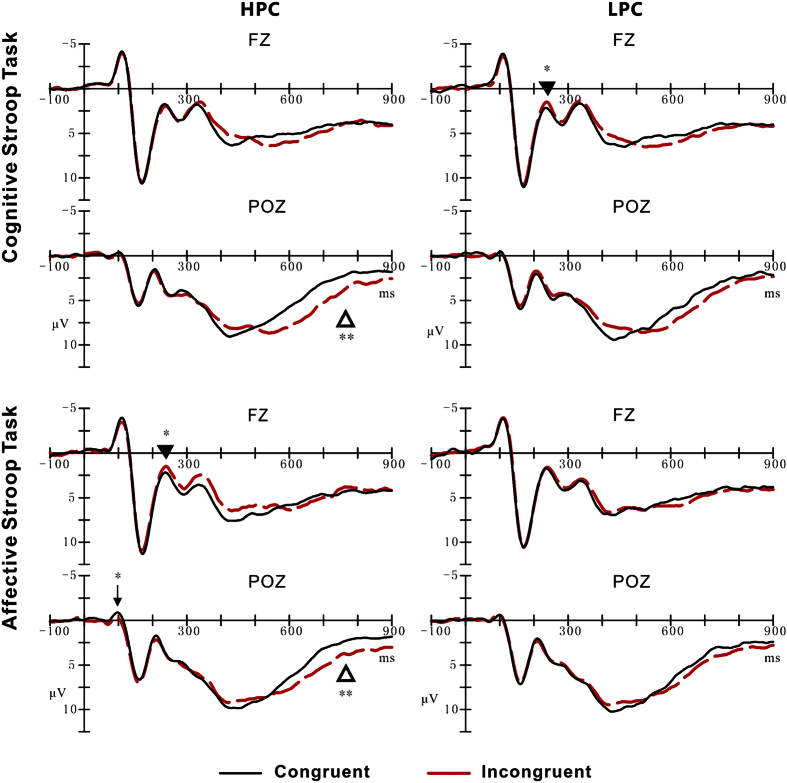
Grand average ERP waveforms at Fz and POz for congruent (black solid lines) and incongruent (red dot lines) trials in the high proportion congruency (HPC) (Left panels) and the low proportion congruency (LPC) (Right panels) contexts during the cognitive task (two top panels) and affective task (two bottom panels). Arrow = N1, Solid triangle = N2, open triangle = SP. *P < 0.05; **P < 0.01.

There were no significant effects on N1 latency over the parieto-occipital areas (*p* > 0.05). For N1 amplitude, there was a significant interaction between Task, Proportion congruency, and Congruency (*F*_1, 21_ = 4.38, *p* < 0.05, *η*^2^ = 0.17). As shown in Table 1 and Fig. 2, larger negative deflections were elicited by congruent compared with incongruent trials only in the HPC context during the affective task (F_1, 21_ = 8.18, *p* < 0.01), while there was no interference effect on N1 amplitude in other conditions (*p* > 0.05). No other significant main effects or interactions involving N1 amplitude were observed (*p* > 0.05).

#### N2

**Figure 3:**
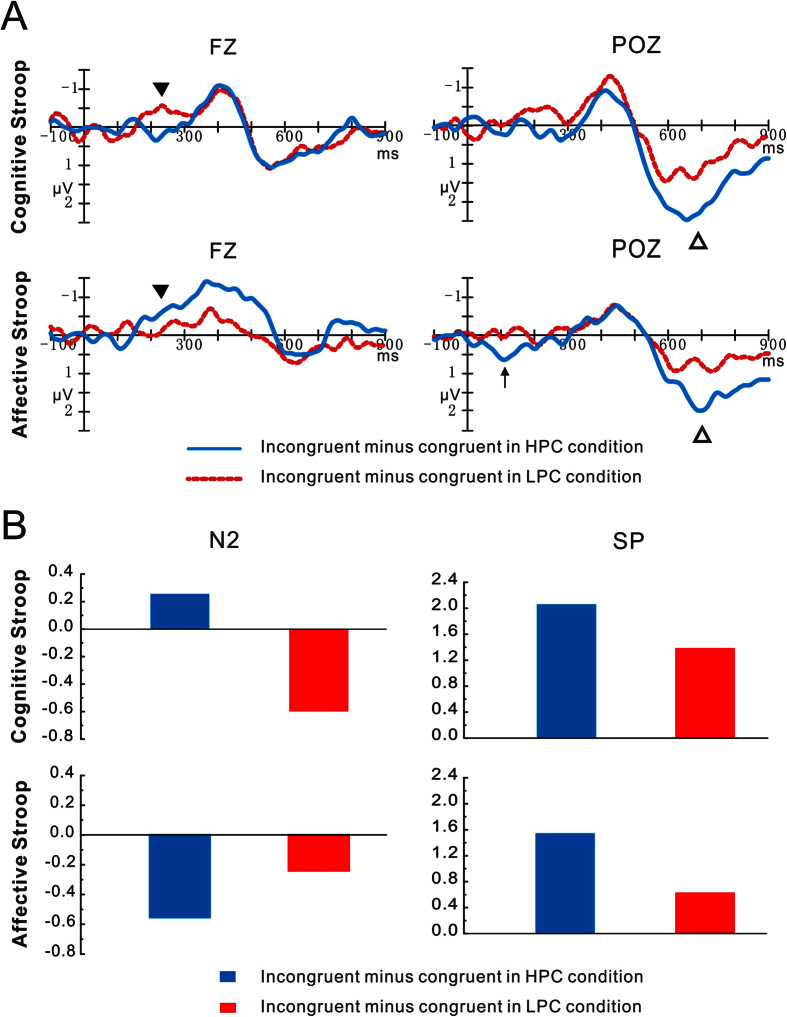
Dissociated effects of congruency context on N2 and SP during cognitive and affective tasks. (**A**) Difference waves at Fz and POz between incongruent and congruent stimuli in the high proportion congruency (HPC) (blue solid lines) and the low proportion congruency (LPC) (red dot lines) context during the cognitive (top panels) and affective tasks (bottom panels). Solid triangle for N2, open triangle for SP. (**B**) Histogram shows the effect of stimulus congruency (i.e., incongruency vs. congruency) on N2 amplitude (μV) (Left panels) and SP amplitude (μV) (Right panels) in the HPC (blue columns) and the LPC (red columns) contexts during the cognitive (top panels) and affective (bottom panels) tasks.

The latency of N2 over the fronto-central areas did not differ significantly across factors (*p* > 0.05). However, as shown in Table 1 and Fig. 2, N2 amplitude showed a marginally significant main effect of Congruency (F_1, 21_ = 3.19, *p* *=* 0.09, *η*^2^ = 0.13), such that larger negative deflections were elicited by incongruent (0.93 μV) than by congruent stimuli (1.19 μV). In addition, a significant interaction between Task, Proportion congruency, and Congruency was identified (F_1, 21_ = 8.62, *p* < 0.01, *η*^2^ = 0.29). Follow-up analyses revealed that larger negative deflections were elicited by incongruent than by congruent trials in the LPC context during the cognitive task (F_1, 21_ = 4.35, *p* < 0.05) and in the HPC context during the affective task (F_1, 21_ = 6.58, *p* < 0.05). In contrast, there was no interference effect in the HPC context during the cognitive task (F_1, 21_ = 1.00, *p* = 0.33) or in the LPC context during the affective task (F_1, 21_ = 0.64, *p* = 0.43). Most importantly, a follow-up analysis computing the interference effect using incongruent minus congruent trials as the dependent variable revealed that the effect was larger in the LPC context (−0.60 μV) than the HPC one (0.30 μV) during the cognitive task, F (1, 21) = 5.62, p < 0.05, but was smaller in LPC context (−0.18 μV) than HPC one (−0.59 μV) during the affective task, F (1, 21) = 4.72, p < 0.05. Thus, the N2 showed an opposite modulation of the PC effect during the cognitive and affective tasks (Fig. 3). As illustrated in Supplementary Fig. 2A, the opposite pattern of the PC effect was also revealed clearly in the N2 voltage maps from the central brain area during the cognitive and affective tasks. No other significant main effects or interactions involving the N2 amplitude were observed (*p* > 0.05).

#### SP

As shown in Table 1 and Fig. 2, SP amplitude exhibited a significant main effect of Congruency (F_1, 21_ = 43.90, *p* < 0.001, *η*^2^ = 0.68), with a larger amplitude being elicited by incongruent trials (4.50 μV) than by congruent ones (3.50 μV). Furthermore, a significant interaction between Congruency and Proportion congruency (F_1, 21_ = 4.55, *p* < 0.05, *η*^2^ = 0.18) was identified. Planned comparisons indicated that a larger SP amplitude was elicited by incongruent trials than by congruent ones in both the HPC context (F_1, 21_ = 28.52, *p* < 0.001, 5.01 μV vs. 3.30 μV) and the LPC one (F_1, 21_ = 26.81, *p* < 0.001, 4.68 μV vs. 3.69 μV). In addition, as shown in Fig. 3, the interference effect on the SP was larger in HPC context than in LPC one (F_1, 21_ = 4.55, *p* < 0.05, *η*^2^ = 0.18; 1.71 μV vs. 1.00 μV). No other significant main effects or interactions on SP amplitude were observed (*p* > 0.05). Supplementary Fig. 2A shows that the positive voltage distributed over the parieto-occipital surface of the skull in the HPC context was stronger than that in the LPC context during both cognitive and affective tasks.

### Correlational analyses

To verify whether ERP components were associated with performance on the conflict tasks, we conducted Pearson’s correlation analyses between conflict-related ERP amplitude and the behavioral response interference in each context. The interference effects of RT were not correlated with N1 or N2 amplitude (*p* > 0.05). However, interference effects of RT were significantly correlated with the conflict-related SP amplitude in the HPC context during both the cognitive (*r* = 0.562, *p* = 0.006) and affective conflict tasks (*r* = 0.555, *p* = 0.007).

### LORETA Results

**Figure 4:**
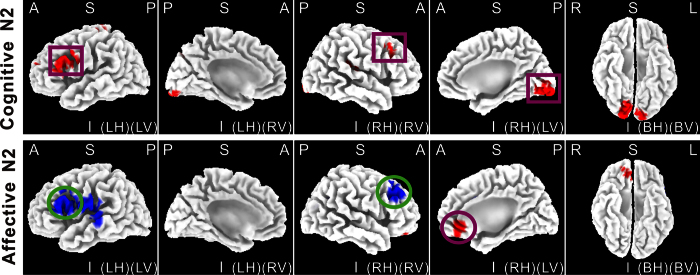
The sLORETA source localization for the difference waves (incongruency minus congruency) of the N2 component between the low and high proportion congruency contexts during cognitive task (top panels) and affective tasks (bottom panels). The image of N2 corresponds to 220 – 280 ms post-stimulus latency. A = anterior. P = posterior. S = superior. I = inferior. LH = left hemisphere. RH = right hemisphere. BH = both hemispheres. LV= left view. RV = right view. BV = bottom view. Red: the maximum activation, Blue: the minimum activation. Square: cognitive task, Circle: affective task.

Figure 4 shows the LORETA solution of the N2 difference wave between the LPC and HPC contexts during cognitive and affective conflict processing. During the cognitive task, the maximum activation was localized at the inferior frontal gyrus (X = 15, Y = −93, Z  = −8, BA 9 and 45), and lingual gyrus (BA 17 and 18). Its minimum activation was localized at the superior temporal gyrus (X = 54, Y = −62, Z = 26, BA39). During the affective conflict task, the maximum activation was localized at the anterior cingulate gyrus (X = 15, Y = 43, Z = −6, BA 32) and medial frontal gyrus (BA 10). Its minimum activation was localized at the middle frontal gyrus (X = 35, Y = 31, Z = 31, BA9).

## Discussion

The primary purpose of the current study was to investigate the influence of PC (i.e., the relative proportion of congruent and incongruent trials) on temporal processing dynamics during cognitive and affective control tasks. Behavior and EEG data were recorded when participants performed cognitive and affective face-word Stroop tasks. We observed comparable interference effects in these two tasks, such that performance of participants was impaired by the incongruent compared to congruent stimuli. Furthermore, our study identified PC modulation of interference effects, in that the behavioral Stroop effect was attenuated in the LPC context compared to the HPC one. HPC context might induce increased global attention for irrelevant words, as well as the predictability of context congruency, leading to enhanced interference effects. In contrast, LPC context might increase focal attention on the task-relevant stimuli (i.e., gender or expressions of faces)^1^, leading to attenuated conflicts.

The PC effect on different stages of cognitive and affective conflict processing was examined with conflict-related ERP components. Firstly, the amplitudes of early N2 and the late SP components were modulated by the PC; secondly, the modulatory effect of PC exhibited opposite patterns on both N2 potentials and their activity generation (i.e., DLPFC) for cognitive and affective tasks. In contrast, similar PC effects were identified at late SP components in the cognitive and affective tasks. These results are consistent with our recent findings^34^ using cognitive and affective flanker tasks, thereby providing two independent demonstrations for the existence of both different and similar temporal dynamics between cognitive and affective conflict processing.

### Dissociable Early Attentional Control Mechanisms Underlying Cognitive and Affective Conflict Processing

In the cognitive word-face Stroop task, a stronger conflict-related N2 effect was observed in the LPC than HPC context, which is consistent with previous studies using similar cognitive conflict tasks^9^,^10^,^34^. The patterns of N2 amplitude in cognitive conflict processing may be attributed to broad attentional and habitual responses to the word and the face components when subjects are expected to process frequent congruent stimuli. Thus, an infrequent incongruent stimulus in the HPC context might elicit sudden and rapid attentional shifts leading to faster responses and an attenuated N2 component. However, when subjects are required to process frequent incongruent stimuli they will predictably and strategically increase their attentional focus on face identification in order to reduce the amount of conflict that they experience. Thus the potential inclination to read the word could be persistently overcome in early conflict processing and this proactive processing eventually benefits response generation, resulting in an enhanced N2 effect in the LPC context^7^,^34^.

However, in the affective Stroop task, the attentional control mechanism may be different due to the prioritizing and permanence of emotional information^22^,^39^,^40^,^41^,^42^. In the HPC context, the facilitation of irrelevant emotional words in frequent congruent trials may reduce the attentional requirement for target responses resulting in enhanced emotional engagement, because more residual attentional resources are focused upon irrelevant the emotional words as well as the relevant emotional faces. Thus, the top-down attentional selection of the target face expression from the ignoring distractor of emotional words in rare incongruent trials may be in competition with ongoing emotional engagement^25^,^26^, resulting in an enhanced N2. This may reflect an increasing attentional demand to resolve the competition between distractor inhibition and affective processing^25^,^26^,^31^. In contrast, in the LPC context during the affective task, most attention resources are initially focused upon the inhibition of the emotional distractor words, and this inhibition persists throughout the entire processing period, even in the congruent trials, resulting in an attenuated N2 effect. Overall, these affective findings suggested the presence of an interaction between emotional processing and top-down attentional selection under the modulation of PC in conflict processing^20^,^43^.

Consistent with previous studies^17^,^18^, the cognitive N2 effects were generated at both DLPFC and sensory cortex. A previous fMRI study has also reported sustained activity of the DLPFC modulated by PC context in a cognitive conflict task^44^. This suggests that conflict with non-emotional stimuli is resolved by the enhancement of a relevant stimulus representation in the sensory cortex followed by top-down control from DLPFC^26^,^45^,^46^. This conclusion is supported by the findings of larger N170 and N300 ERP components in the cognitive conflict task (see more details in Supporting Materials), suggesting that increased attentional resources were engaged in face perception and categorization during the face gender identification task^47^,^48^,^49^. In contrast, during the affective conflict task N2 effects were generated at DLPFC and rACC suggesting that increased attentional resources were engaged in inhibition of face expression identification during the task^50^,^51^. Importantly, the opposite PC effects on N2 amplitude in cognitive and affective tasks were localized in the DLPFC, with activity increased in the cognitive task but reduced in the affective one. Overall, these findings cannot be explained by the dominant conflict control theory^12^ which proposes that dACC is activated first and drives DLPFC in cognitive control and that dACC drives rACC in affective control^26^ when conflict is increased. A novel and more integrated hypothesis which can incorporate both the similarities and differences between neural processing of cognitive and affective conflict is therefore needed.

Notably, a more negative N1 component was evoked by congruent than incongruent trials in the HPC context during the affective task, which is consistent with our previous study^34^. This is an interesting finding which may reflect variable attentional engagement in relation to emotional information in the HPC context. The most likely interpretation of the attentional mechanism is that there are effects of perceptual or attentional additivity involved when individuals pay attention to facial affect, see congruent information, and have many congruent trials to learn this congruency and respond to it visually. This supports our proposal that emotionally-induced additivity may compete with conflict detection for the rare incongruent trials which produce larger N2 effects in the HPC context in the affective task. Alternatively, such changes might reflect the complexity of attentional selection for emotional information under low attentional focus, a possibility that needs to be clarified in future studies using different manipulations of attentional anticipation.

### Similar Late Response System Associated With Cognitive and Affective Control

In the present study, the SP component showed similar patterns of modulation by the PC effect during cognitive and affective tasks, which is also consistent with observations in our previous study^34^. Moreover, the SP effect correlated positively with response interference only in the HPC context during both tasks, suggesting that the SP may represent the response stage of conflict processing^13^,^19^,^34^,^52^. This conjecture echoes topographical findings showing a positive voltage distributed over the parieto-occipital surface of the skull since the SP was positively activated in the inferior parietal lobe during the two tasks (see more details of the discussion on SP localization in Supporting Materials). The inferior parietal lobe may be involved in controlling goal-directed behaviors and execution of action^53^,^54^,^55^. Our current experimental findings therefore directly support the widely held view that the SP reflects conflict resolution in cognitive^13^,^14^,^19^,^56^ and affective^34^,^57^ conflict tasks.

## Conclusion

In summary, in addition to the PC effects on behavior demonstrated using Stroop tasks, the current study also found different early attentional processing but similar late response processing for cognitive and affective conflict tasks involving N2 and SP evoked potential components respectively. These results suggest that cognitive and affective controls share a similar conflict response system but dissociable early attentional control mechanisms.

## Material and Methods

### Participants

Twenty-two young healthy adults (10 females, mean age = 21.80 years, SD = 1.91 years, range 19–25 years) were paid to participate in this study. All subjects were right handed and had normal or corrected to normal vision by self-report. None had any reported history of neurological or psychiatric diseases. The research protocol was approved by the ethics committee of Beijing Normal University. Written informed consent was obtained from all participants prior to the study, which was approved by the Institutional Review Board of Beijing Normal University Imaging Center for Brain Research. The methods were conducted in accordance with approved guidelines.

### Stimulus material and experimental task

A total of 32 human face pictures, including 16 (8 females and 8 males) depicting a happy expression and 16 (8 females and 8 males) depicting a fear expression, were selected from the Chinese Affective Picture System^58^. Participants performed two modified versions of face-word Stroop tasks. In the cognitive face-word Stroop task, faces were presented with either the Chinese word “
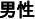
” (“nanxing”, means male) or “
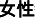
” (“nǚxing”, means female) superimposed across the face (Fig. 1A, top panels), producing gender-congruent and -incongruent stimuli^26^. In the affective face-word Stroop task, faces were presented with the Chinese word “
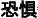
” (“kongju”, means fear) or “
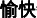
” (“yukuai”, means happy) superimposed across the face (Fig. 1A, bottom panels), such that the word and facial expression were either congruent or incongruent^26^,^59^. Participants were required to categorize the gender or expression of faces while trying to ignore the task-irrelevant word stimuli. The words were in red and projected approximately across the center of the faces (i.e. across the nose region). The size of the Chinese characters in bold was about 1° (horizontal) × 1° (vertical).

### Experimental procedure

All participants performed both the cognitive and affective Stroop tasks, with the order of tasks being counterbalanced across subjects. Each task consisted of three sessions, and each session included four blocks with two kinds of PC: two blocks with a HPC context consisting of 70% congruent and 30% incongruent trials; and two blocks with a LPC context consisting of 70% incongruent and 30% congruent trials (Fig. 1B). Each block consisted of 52 randomly presented trials. The two PC contexts were presented in an ABBA or BAAB order which was counterbalanced across subjects. Half of the participants responded to the fearful or male faces with the index finger and to the happy or female faces with the middle finger of their right hand, and the opposite mapping was used for the other half of the participants. Consistent with our previous study^34^, each trial began with a 1500 ms fixation (white cross) followed by a 1500 ms photographic stimulus (3.5° wide and 5° high) on the center of the black screen. During the presentation of stimuli, participants were instructed to respond as quickly and accurately as possible. The stimulus disappeared once a subject’s response was made. All participants achieved above 85% accuracy on the 20 practice trials prior to the formal experiment. The tasks were programmed in E-Prime (Psychology Software Tools, Inc.) and run using a Hewlett-Packard (HP) Pavilion f523 computer with a 17- inch color CRT monitor.

We controlled for: (i) stimulus repetition^60^ by using alternative target stimuli across trials (i.e., a face picture would not be repeated in the following trial), (ii) response repetitions^26^ by employing alternate responses to target stimuli across trials (i.e., a response to a face picture would not be repeated in the following trial) and keep the same proportion (50%) of response alternations to target faces for all congruency trial types, and (iii) negative priming^61^ by avoiding direct repetitions of the same face with varying word distracters (i.e., the distractor word in the present trial would be different with the response to a target face judgment in the following trial). Furthermore, there was no category switch cost^62^ on the time course of cognitive and affective conflict processing (see also Supplemental Methods and Discussion for more details about the control analysis)

### Electrophysiological data recording and processing

The electroencephalogram (EEG) was recorded from 64 scalp sites using tin electrodes mounted in an elastic cap (NeuroScan Inc., Herndon, Virginia, USA) according to the international 10/20 system. The left mastoid was used as reference electrode. All electrode impedances were below 5 kΩ. The EEG was online sampled at an A/D rate of 500 Hz/channel and a band-pass of 0.05–100 Hz. A 30 Hz low-pass filter was used offline. Trials with signals exceeding ± 80 μV were automatically excluded from the average. See Supplemental Methods for more details about the recording procedure.

### Data analysis

Error trials, post-error trials and the first trial of each block were excluded from analyses of both the RTs and ERP data. This cut-off procedure excluded 9.8% of all the trials. The number of epochs included in the ERP averages was above 75 for each condition.

We selected the time window and electrode sites for different components on the basis of (i) previous relevant studies^11^,^34^, (ii) visual inspection of the topographical distribution of grand averaged ERPs, and (iii) difference waves for each subject. The following components were analyzed: N1 (80–150 ms) at CP3, CPz, CP4, P3, Pz and P4 electrode sites, N2 (220–280 ms) at F3, Fz, F4, FC3, FCz and FC4 electrode sites, as well as the conflict SP (650–750 ms) at P3, Pz, P4, PO3, POz and PO4 electrode sites. In light of previous studies, the peak latencies (time duration from stimulus onset to the peak of each component) and baseline-to-peak amplitude were measured and analyzed for N1 and N2 components, while mean amplitude was measured and analyzed for SP to make current results comparable to previous findings^11^,^34^. To compare the time course of cognitive and affective conflict processing, we also analyzed N170, P1, N300 and P300, the main task specific ERP components^47^,^48^,^49^ (see Supplemental Methods and Discussion for more details about the analysis of task specific components). Segments of 100 ms before and 900 ms after the onsets of stimuli were extracted for each component from the continuous EEG, and the pre-stimulus baseline was removed.

A 3-way repeated measures analysis of variance (ANOVA) on the amplitude and latency of each component was conducted with following independent variables: Task (cognitive, affective), Proportion congruency (high, low), Congruency (congruent, incongruent). The dependent variables were the average values of all electrode sites selected for each ERP component. Bonferroni correction for the p-values was used to control for multiple comparisons, and *p* values were corrected by Greenhouse–Geisser if necessary. Differences were considered significant at *p* < 0.05, and partial-eta^2^ (*η*^2^) is reported as a measure of effect size. All statistical analyses were carried out with SPSS (Version 17.0, Chicago, SPSS Inc.).

### LORETA source localization method

In order to investigate the localization of the generators of PC effects during conflict processing, we carried out an sLORETA analysis using differences in the ERP components^63^. This method has no localization bias and provides a genuine inverse solution with exact, zero error localization. It is capable of imaging standardized current density with nearly zero localization error. The electrical potential lead field was calculated using the boundary element method^64^. Electrode coordinates were registered to the digitized Montreal Neurological Institute (MNI) standard brain^65^. Talairach space was used to represent the electrical activity of each voxel^66^. The cortical surface was based on Van Essen average cortex^67^. For details of the sLORETA analysis, see also^34^ and Supplemental Methods.

## Additional Information

**How to cite this article**: Chen, T. *et al*. Dissociable early attentional control mechanisms underlying cognitive and affective conflicts. *Sci. Rep.* **6**, 37633; doi: 10.1038/srep37633 (2016).

**Publisher's note:** Springer Nature remains neutral with regard to jurisdictional claims in published maps and institutional affiliations.

## Supplementary Material

Supplementary Information

## Figures and Tables

**Table 1:** Mean latencies (ms) and amplitude (μV) of N1, N2 and SP components elicited by the congruent and incongruent stimuli in the high and low proportion congruency contexts during cognitive and affective Stroop tasks. Standard deviations are shown in brackets.

		Cognitive Stroop task				Affective Stroop task			
		HPC		LPC		HPC		LPC	
		Latency	Amplitude	Latency	Amplitude	Latency	Amplitude	Latency	Amplitude
**N1**	Congruent	106 (9)	−2.31 (2.09)	103 (11)	−2.39 (2.15)	103 (9)	−2.36 (2.17)	103 (11)	−2.38 (1.83)
(80–150 ms)	Incongruent	104 (10)	−2.43 (2.44)	102 (10)	−2.13 (2.08)	101 (12)	−1.91 (1.98)	103 (9)	−2.32 (2.03)
**N2**	Congruent	245 (20)	0.96 (3.60)	241 (17)	1.41 (4.06)	239 (17)	1.38 (3.81)	241 (19)	1.03 (3.91)
(220–280 ms)	Incongruent	244 (19)	1.25 (3.64)	242 (17)	0.81 (3.63)	241 (17)	0.79 (3.98)	241 (18)	0.85 (4.09)
**SP**	Congruent		3.15 (3.46)		3.61 (3.32)		3.45 (2.29)		3.77 (2.72)
(650–700 ms)	Incongruent		5.08 (4.02)		4.85 (3.39)		4.95 (3.35)		4.52 (2.90)

Note: high proportion congruency, HPC; low proportion congruency, LPC.
