# Supplementary material for: Dissociable early attentional control mechanisms underlying cognitive and affective conflicts
Source: Sci Rep. 2016 Nov 28;6:37633. doi: 10.1038/srep37633 (PMC5124857; doi:10.1038/srep37633)
Supplement: Supplementary Information [file srep37633-s1.doc]

**Dissociable early attentional control mechanisms underlying cognitive and affective conflicts**

**Taolin Chen, Keith M. Kendrick, Chunliang Feng, Shiyue Sun, Xun Yang, Xiaogang Wang, Wenbo Luo, Suyong Yang, Xiaoqi Huang, Pedro A. Valdés-Sosa, Qiyong Gong, Jin Fan and Yuejia Luo**

**Supporting Material**

**Supplemental Methods**

**Electrophysiological data recording**

Participants were seated in a comfortable chair in an electrically shielded room with attenuated sound and lighting levels and were instructed to look at the center fixation point on the screen in front of them and not to move during the recording session. Participants were grounded with a forehead electrode. All EEG channels were referenced to the left mastoid, and were re-referenced offline to the average of the left and right mastoids by subtracting from each sample of data recorded at each channel one-half the activity recorded at the right mastoid. A horizontal electrooculogram (EOG) was recorded from electrodes placed at the outer canthi of both eyes and the vertical EOG was recorded from electrodes placed above and below the left eye. Ocular artifacts were corrected off-line with an algorithm [^1^](#_ENREF_1). The EEG data was preprocessed using the EDIT program from the software package SCAN (Version 4.3, NeuroScan Inc., Herndon, Virginia, USA).

**Task specific ERP components analysis**

The following task specific components were analyzed in the present study: N170 (150 – 200 ms) at P7, P8, PO7and PO8 electrode sites, N300 (280 – 350 ms) at F3, Fz, F4, FC3, FCz and FC4 electrode sites, P1 (90 – 170 ms) at O1, O2, PO5 and PO6 electrode sites, and P300 (350 – 550 ms) at CP3, CPz, CP4, P3, Pz and P4 electrode sites. Based on the inspection of averaged waves of the ERP components and the previous studies [^2-5^](#_ENREF_2), the peak latencies (time duration from stimulus onset to the peak of each component) and baseline-to-peak amplitudes were measured and analyzed for N170, N300, P1, and P300 components.

**LORETA source localization method**

The processing steps of source localized analysis are consistent with our previous study [^4^](#_ENREF_4) and according to the method of [^6^](#_ENREF_6). In brief, we firstly computed the topographic analysis of variance (TANOVA) to assess non-parametrically each pair of topographic ERP maps of scalp potentials within each condition. The periods where topographic ERP maps showed statistical significance between each of the conditions, such as the difference waves of incongruent minus congruent trials in the frequent incongruent context vs. that in the frequent congruent context in the present study were denoted as “time segments”. These time segments were evaluated using univariate, paired-samples t-test, computed for their amplitudes and log-transformed LORETA values (*p* < .05).

In a second step we computed the source localization of observed differences. Localization computation was based on voxel-by-voxel t-tests of LORETA images between the difference waves in frequent incongruent context vs. that in frequent congruent context. The LORETA images were obtained from the average of current density magnitude over all instantaneous LORETA images for each voxel within a given time segment. A single null hypothesis was tested for significance by means of a voxel-by-voxel t-test at *p* < .05. Overall statistical significance was evaluated non-parametrically using a randomization test which corrects for multiple comparisons.

**Supplemental ERP results**

***P1***

The latency of P1 over the occipital areas showed a significant main effect of task type (F_1, 21_ = 8.24, *p <* 0.01, *η*^2^ = 0.28). As shown in Supplementary Table 1 and Supplementary Figure 1, the latency during the affective task was longer than that during the cognitive one (139 ± 3.60 ms vs. 133 ± 3.44 ms). No other significant main effects or interactions on latency were observed (*p >* 0.05). P1 amplitudes showed a significant main effect of task type (F_1, 21_ = 4.69, *p <* 0.05, *η*^2^ = 0.18), since larger amplitudes were elicited during the affective compared with the cognitive task (5.19 ± 0.63 μV vs. 4.55 ± 0.63 μV). No other significant main effects or interactions involving amplitude were observed (*p >* 0.05).

***N170***

As shown in Supplementary Table 1 and Supplementary Figure 1, there was no significant difference in N170 latency across factors (*p >* 0.05). However, N170 amplitudes showed a significant main effect of task type (F_1, 21_ = 9.83, *p <* 0.01, *η*^2^ = 0.32). Larger negative deflections were elicited during the cognitive task (− 2.22 ± 0.73 μV) than during the affective one (− 1.53 ± 0.66 μV). No other significant main effects or interactions involving amplitude were observed (*p >* 0.05).

***N300***

No significant N300 latency difference was observed (*p >* 0.05). However, as shown in Supplementary Table 1 and Supplementary Figure 1, N300 amplitudes revealed significant main effects of congruence (F_1, 21_ = 8.74, *p <* 0.01, *η*^2^ = 0.30) and task type (F_1, 21_ = 7.33, *p <* 0.05, *η*^2^ = 0.26). Larger negative deflections were elicited by incongruent than by congruent trials (1.52 ± 1.17 μV vs. 1.99 ± 1.15 μV) and during the cognitive compared with the affective task (1.22 ± 1.09 μV vs. 2.30 ± 1.25 μV). No other significant main effects or interactions on N300 amplitude were observed (*p >* 0.05).

***P300***

As shown in Supplementary Table 1 and Supplementary Figure 1, P300 latency exhibited a significant main effect of congruence (F_1, 21_ = 16.92, *p <* 0.001, *η*^2^ = 0.45) with longer latencies being elicited by incongruent than by congruent trials (481.83 ± 9.23 ms vs. 464.80 ± 7.95 ms). No other significant main effects or interactions on P300 latency were observed (*p >* 0.10). P300 amplitudes showed a significant main effect of congruence (F_1, 21_ = 11.79, *p <* 0.01, *η*^2^ = 0.36) and a marginally significant main effect of task type (F_1, 21_ = 4.23, *p=* 0.05, *η*^2^ = 0.17). Larger amplitudes were elicited by congruent than incongruent trials (11.66 ± 1.13 μV vs. 11.14 ± 1.16 μV) and during the affective compared with the cognitive task (11.82 ± 1.22 μV vs. 10.98 ± 1.10 μV). No other significant main effects or interactions on P300 amplitude were observed (*p >* 0.05).

**Control analysis: whether category switch cost influenced conflict control**

To test whether switching face category had an impact on conflict interference and modulated the effect of task context on cognitive and affective control, we separated sequential trials repeating the same category (e.g. from female to female or male to male in the cognitive task and from happy to happy or fear to fear in the affective task) from those changing to a different category (e.g. from female to male or male to female in the cognitive task and from happy to fear or fear to happy in the affective task). A four way task type × switch × block type × electrode site (as within-subjects factors) repeated measures ANOVA was conducted on the mean latency and amplitude of the ERP during the cognitive and affective tasks. As illustrated in Supplementary Figure 3, the N300 latency showed a significant main effect of task type (F_1, 21_ = 5.10, *p <* 0.05, *η*^2^ = 0.20) and of switch (F_1, 21_ = 9.27, *p <* 0.05, *η*^2^ = 0.31), due to a longer latency being elicited during the cognitive than during the affective task (323.20 ± 3.70 ms vs. 314.37 ± 4.28 ms) and by switching stimulus category rather than it staying the same (320.92 ± 3.67 ms vs. 316.65 ± 3.45 ms). There was a significant interaction between switching and block type (F_1, 21_ = 4.88, *p <* 0.05, *η*^2^ = 0.19), since stimulus switching elicited a significantly longer latency than when it stayed the same in the low proportion congruency (LPC) context (F_1, 21_ = 8.65, *p <* 0.01) rather than in the high proportion congruency (HPC) context (F_1, 21_ = 2.24, *p =* 0.15). N300 amplitudes showed a significant main effect of task type (F_1, 21_ = 8.56, *p <* 0.01, *η*^2^ = 0.30) and stimulus switching (F_1, 21_ = 11.01, *p <* 0.01, *η*^2^ = 0.34), as larger negative amplitudes were elicited during the cognitive than during the affective task (1.10 ± 1.07 μV vs. 2.27 ± 1.25 μV), and for switched stimuli as opposed to ones that stayed the same (1.41 ± 1.14 μV vs. 1.96 ± 1.15 μV). No other significant main effects or interactions on N300 latency or amplitude were found (*p >* 0.05). The above results show that the cost of switching was larger in the cognitive than the affective task. Only the latency of the N300 was influenced by block type during the two tasks.

**LORETA Results**

Supplementary Figure 4 shows the LORETA solution for the SP effect between the high and low proportion congruency contexts during cognitive and affective conflict processing. During the cognitive task, the maximum activation was localized at (X = 45, Y = 21, Z = 36), having a best match in the Talairach atlas at Brodmann areas (BA) 9 and 46 (middle frontal gyrus) and at BA 40 (inferior parietal lobe), as well as at BA 17 (lingual gyrus). However, its minimum activation was localized at (X = - 5, Y = 36, Z = 40) with a best match at BA 8 (medial frontal gyrus) and BA6 and 30 (cingulate gyrus). During the affective conflict task, the maximum activation was localized at (X = - 15, Y = 43, Z = - 23) with best matches at BA 11 and 47 (orbital gyrus) and at BA 40 (inferior parietal lobule). However, its minimum activation was localized at (X = - 64, Y = - 48, Z = 12) with best matches at BA 22 and 21 (superior temporal gyrus) and BA 9 (middle frontal gyrus).

**Supplemental Discussion**

**Task-specific Processing Associated with Cognitive and Affective Conflict**

Regardless of the modulation of congruency context, the differences and similarities in the temporal dynamics of both versions of conflict tasks were observed in the present study. Larger amplitudes of P1 and P300 were elicited during the affective task compared with cognitive tasks. P1 reflects an early attention-related neural processing to emotional stimuli and the P300 is thought to reflect stimulus evaluation during cognitive conflict tasks [^11^](#_ENREF_11). These findings support the view that emotional information induces an increase in more automatic aspects in conflict processing [^12^](#_ENREF_12) and an enhanced inhibition of emotional information during the face expression evaluation processing .

In addition, larger amplitudes of N170 and N300 were elicited during the cognitive task compared with affective one. The N170 reflects the early processing of facial perception [^2^](#_ENREF_2) and the N300 is thought to represent picture categorization . The larger N170 and N300s in the cognitive conflict task suggest that increased attentional resources were engaged in the object perception and categorization during the face gender identification task . Our findings provide further evidence to support the view that attentional target-feature amplification is the primary mechanism for conflict resolution through cognitive control .

**Absence of Category Switch Cost on Cognitive and Affective Conflict**

To examine whether category switching of target face stimuli had a cost in terms of cognitive and affective interference, and whether conflict context would influence this cost, we reanalyzed and compared sequential trials with the same category of face (non-switch category trial) with those having a different one (switch category trial). We found a frontal N300 component during the cognitive and affective tasks with a larger amplitude during switch than non-switch trials. Interestingly, we also found another N300 component in the analysis of conflict interference. Comparing the N300 in the switch analysis with that in the conflict analysis, they appear at the same peak latency (320-330 ms) and have similar kinds of main effects. Both were larger during the cognitive than during the affective task and in the high attentional demand (incongruent trials or switch trials) compared with the low attentional demand trials (congruent trials or non-switch trials) across tasks. Moreover, we did not find any switch cost effect on either the early or late conflict potentials in the high and low proportion congruency contexts during the tasks. This suggests that the N300 is an independent component reflecting face picture categorization and therefore category switching is not associated with a cost during cognitive and affective interference. Moreover, because the N300 amplitude was not modulated by task contexts, it might possibly be considered as a separate component in between the early and late control processing stages within cognitive and affective conflict.

**Localization of SP**

Consistent with the topographical findings showing a positive voltage distributed over the parieto-occipital surface of the skull, the SP was positively activated in the inferior parietal lobe during the two tasks. The inferior parietal lobe may be involved in controlling goal-directed behaviors and execution of action [^19-21^](#_ENREF_19). The SP was also activated in other brain areas including the superior temporal lobe and cingulate gyrus, as well as the same brain areas localized by the early N2 effect during the two tasks. Thus, our results provide evidence for involvement of a late conflict processing stage supporting the view that affective and cognitive control may involve a more complex mechanism than response execution for changing conflict context [^22^](#_ENREF_22).

| **Supplementary Table 1.** Mean latencies (ms) and amplitudes (μV) of P1, N170, N300 and P300 components elicited by the congruent and incongruent stimuli in the high and low proportion congruency contexts during cognitive and affective Stroop tasks. Standard deviations are shown in brackets. | | | | | | | | | | | | | |  |
| --- | --- | --- | --- | --- | --- | --- | --- | --- | --- | --- | --- | --- | --- | --- |
|  | |  | Cognitive Stroop task | | | | |  | Affective Stroop task | | | | |  |
|  | |  | HPC | |  | LPC | |  | HPC | |  | LPC | |  |
|  | |  | Latency | Amplitude |  | Latency | Amplitude |  | Latency | Amplitude |  | Latency | Amplitude |  |
| **P1** | | Congruent | 133(18) | 4.44(2.96) |  | 134(16) | 4.62(2.86) |  | 139(17) | 4.96(2.89) |  | 140(17) | 5.27(3.19) |  |
| (90-170ms) | | Incongruent | 131(17) | 4.53(3.05) |  | 134(18) | 4.63(3.07) |  | 140(17) | 5.3(2.79) |  | 139(18) | 5.25(3.06) |  |
|  | |  |  |  |  |  |  |  |  |  |  |  |  |  |
| **N170** | | Congruent | 174(14) | -2.25(3.37) |  | 174(15) | -2.03(3.24) |  | 172(16) | -1.64(3.23) |  | 173(15) | 1.48(3.09) |  |
| (150-200ms) | | Incongruent | 173(13) | -2.49(3.72) |  | 174(13) | -2.11(3.48) |  | 173(14) | -1.53(3.19) |  | 173(16) | 1.46(3.14) |  |
|  | |  |  |  |  |  |  |  |  |  |  |  |  |  |
| **N300**  (280-350ms) | | Congruent | 321(19) | 1.33(5.18) |  | 325(19) | 1.42(5.18) |  | 316(24) | 2.83(5.84) |  | 317(26) | 2.38(5.79) |  |
|  |  | Incongruent | 324(23) | 1.12(5.27) |  | 326(21) | 0.99(5.03) |  | 318(26) | 1.81(5.97) |  | 316(27) | 2.15(6.05) |  |
|  | |  |  |  |  |  |  |  |  |  |  |  |  |  |
| **P300** | | Congruent | 459(50) | 11(5.31) |  | 462(45) | 11.28(5.17) |  | 476(48) | 12.2(5.31) |  | 462(45) | 12.17(5.94) |  |
| (350-550ms) | | Incongruent | 488(53) | 10.91(5.27) |  | 482(55) | 10.72(5.03) |  | 484(49) | 11.45(5.87) |  | 473(56) | 11.47(6.08) |  |
|  | Note: high proportion congruency, HPC; low proportion congruency, LPC. | | | | | | | | | | | | | |


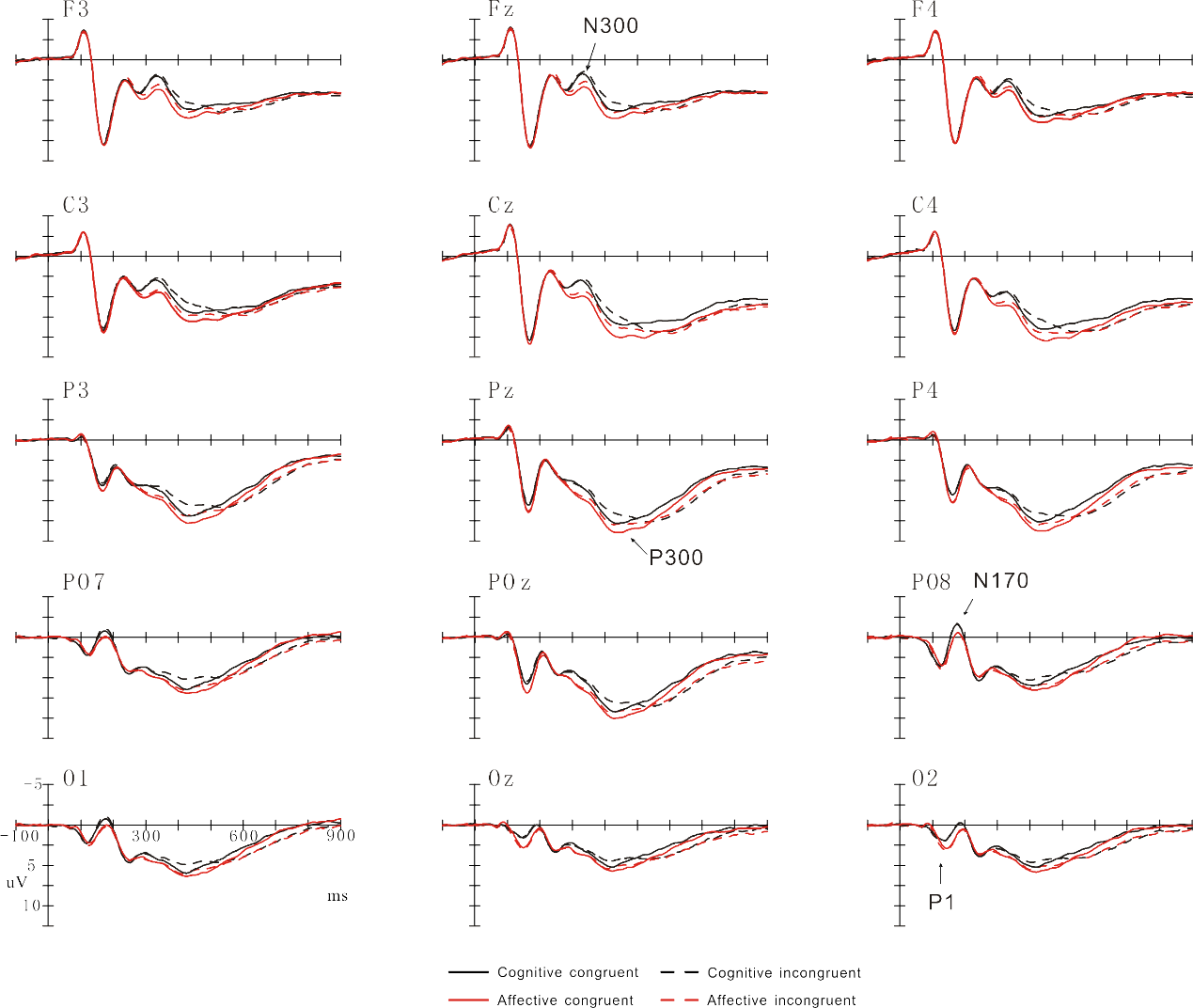


**Supplementary Figure 1.** Grand average ERP waveforms of N170, P1, N300, P300 components for congruent (black solid lines) and incongruent (black dot lines) trials during the cognitive tasks, and for congruent (red solid lines) and incongruent (red dot lines) trials during the affective tasks.


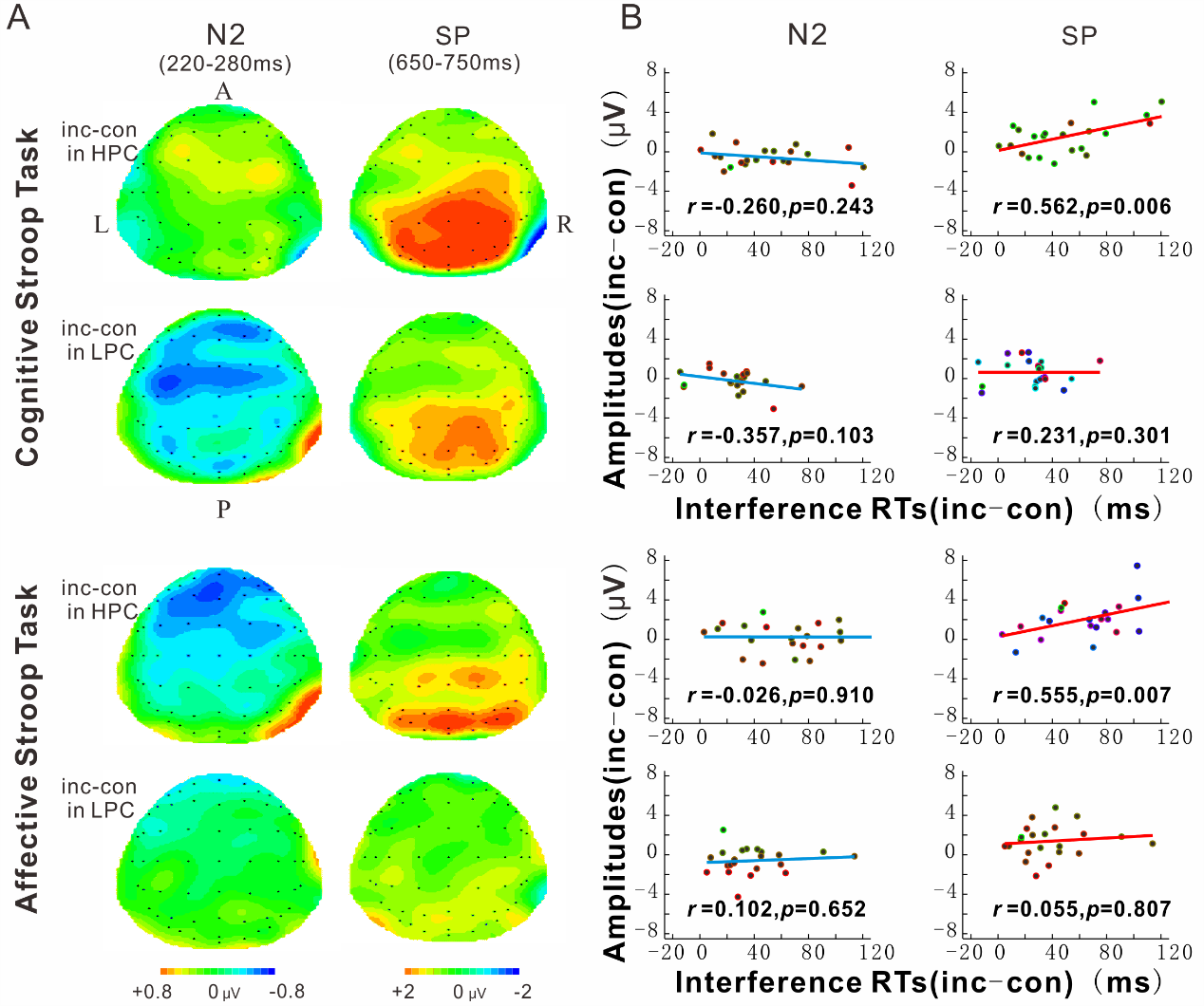


**Supplementary Figure 2.** Dissociable voltage maps of the N2 components in the high proportion congruency (HPC) and low proportion congruency (LPC) contexts during both cognitive and affective tasks. (A) The voltage maps showing the topographical distribution of the congruity effect amplitude (μV) of the N2 (220 – 280ms, left panels) and SP (650-750ms, right panels) components in the HPC context (upper panels) and the LPC context (below panels) during the cognitive (two top panels) and affective tasks (two bottom panels). (B) Pearson’s correlations (two-tailed) between the incongruent minus congruent difference waves for both the N2 and conflict SP components and incongruent minus congruent RTs in the HPC (upper panels) and the LPC contexts (lower panels) during the cognitive (two top panels ) and affective tasks (two bottom panels). Head seen from above, left ear left; L = left, R = right, A= anterior, P = posterior, red = positive potential, blue = negative potential.


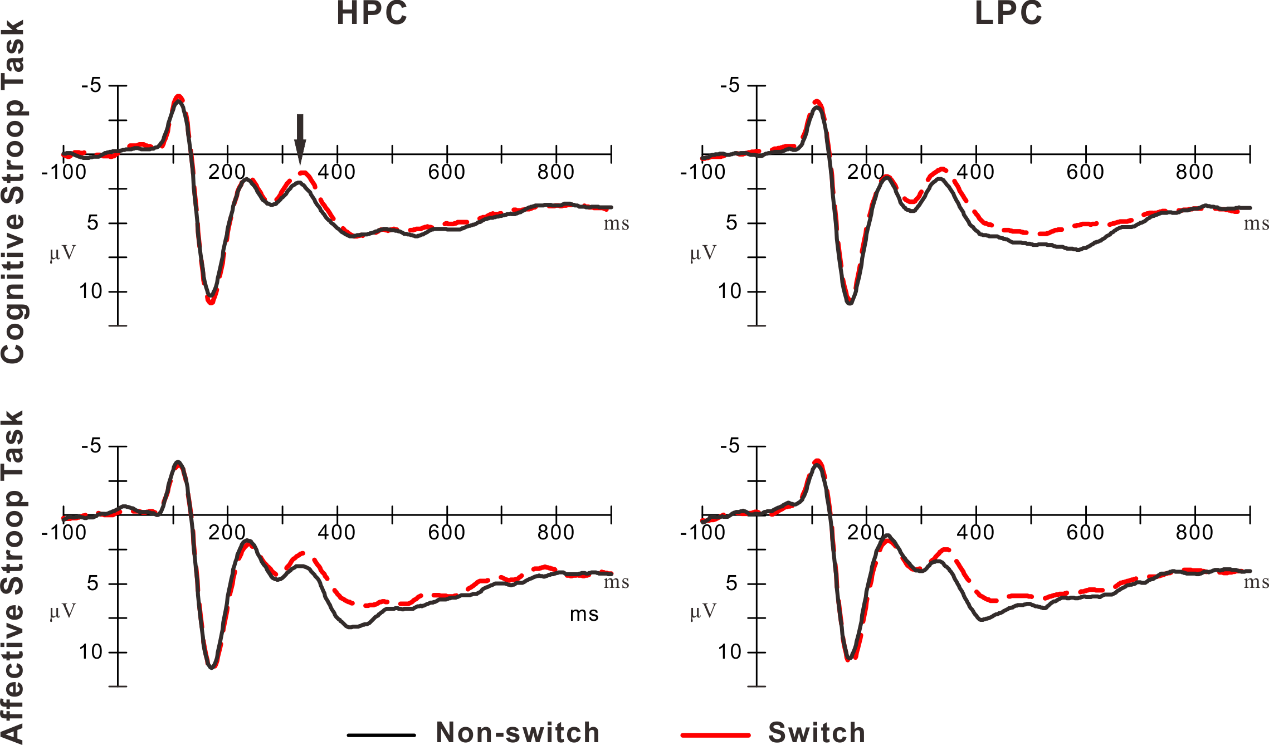


**Supplementary Figure 3.** Grand average ERP waveforms at Fz for non-switch (black solid line) and switch (red dot line) trials in the high proportion congruency (HPC, Left panels) and the low proportion congruency (LPC, Right panels) context during the cognitive (top panels) and affective Stroop tasks (bottom panels). Arrow = N300.


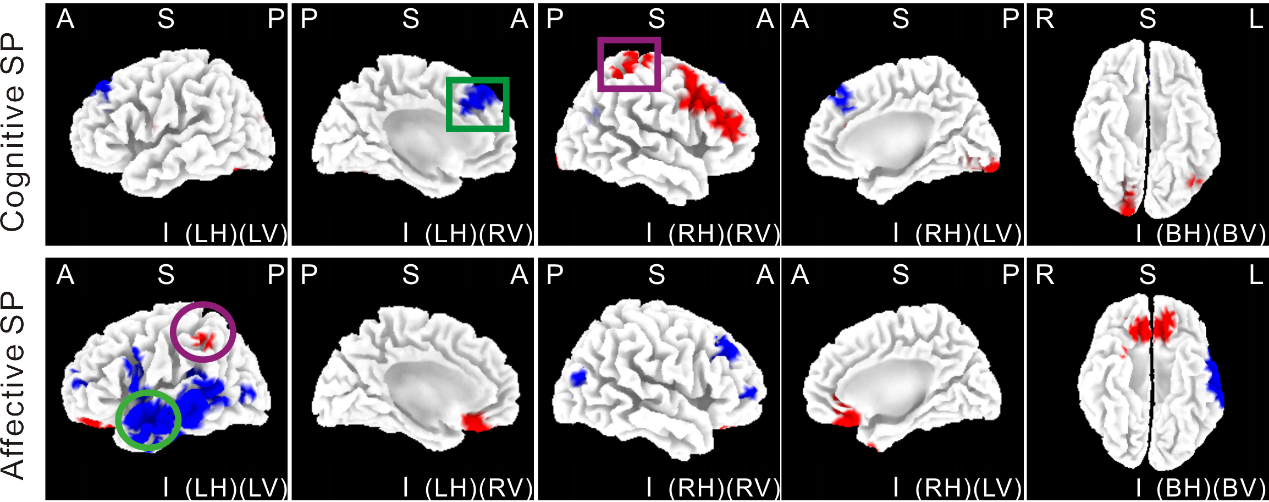


**Supplementary Figure 4.** The sLORETA source localization for the difference waves (incongruency minus congruency) of the SP component between the low and high proportion congruency contexts during cognitive task and affective tasks. The image of SP corresponds to 650-750ms post-stimulus latency. A= anterior. P = posterior. S = superior. I = inferior. LH = left hemisphere. RH = right hemisphere. BH = both hemispheres. LV= left view. RV = right view. BV = bottom view. Red: the maximum activation, Blue: the minimum activation. Square: cognitive task, Circle: affective task.

**Supplemental References**

**1.** Semlitsch HV, Anderer P, Schuster P, Presslich O. A solution for reliable and valid reduction of ocular artifacts, applied to the P300 ERP. *Psychophysiology* **23**, 695-703 (1986).

**2.** Bentin S, Allison T, Puce A, Perez E, McCarthy G. Electrophysiological studies of face perception in humans. *Journal of cognitive neuroscience* **8**, 551-565 (1996).

**3.** Chen, et al. The timing of cognitive control in partially incongruent categorization. *Human brain mapping* **29**, 1028-1039 (2008).

**4.** Chen T., et al. Opposite effect of conflict context modulation on neural mechanisms of cognitive and affective control. *Psychophysiology* **51**, 478-488 (2014).

**5.** Luo W, Feng W, He W, Wang N-Y, Luo Y-J. Three stages of facial expression processing: ERP study with rapid serial visual presentation. *NeuroImage* **49**, 1857-1867 (2010).

**6.** Pascual-Marqui RD. Standardized low-resolution brain electromagnetic tomography (sLORETA): Technical details. *Memory & Cognition* **24**, 5-12 (2002).

**7.** Rogers RD, Monsell S. Costs of a predictable switch between simple cognitive tasks. *Journal of Experimental Psychology: General* **124**, 207-231 (1995).

**8.** Verguts T, Notebaert W. Adaptation by binding: a learning account of cognitive control. *Trends in cognitive sciences* **13**, 252-257 (2009).

**9.** Moser JS, Huppert JD, Duval E, Simons RF. Face processing biases in social anxiety: An electrophysiological study. *Biological psychology* **78**, 93-103 (2008).

**10.** Kanske P, Kotz SA. Modulation of early conflict processing: N200 responses to emotional words in a flanker task. *Neuropsychologia* **48**, 3661-3664 (2010).

**11.** Kutas M, McCarthy G, Donchin E. Augmenting mental chronometry: the P300 as a measure of stimulus evaluation time. *Science* **197**, 792-795 (1977).

**12.** Thomas SJ, Johnstone SJ, Gonsalvez CJ. Event-related potentials during an emotional Stroop task. *International Journal of Psychophysiology* **63**, 221-231 (2007).

**13.** Clayson PE, Larson MJ. Conflict adaptation and sequential trial effects: support for the conflict monitoring theory. *Neuropsychologia* **49**, 1953-1961 (2011).

**14.** McPherson WB, Holcomb JB. An electrophysiological investigation of semantic priming with pictures of real objects. *Psychophysiology* **36**, 53-65 (1999).

**15.** Hamm JP, Johnson BW, Kirk IJ. Comparison of the N300 and N400 ERPs to picture stimuli in congruent and incongruent contexts. *Clinical Neurophysiology* **113**, 1339-1350 (2002).

**16.** Eimer M. Effects of face inversion on the structural encoding and recognition of faces. Evidence from event-related brain potentials. *Cognitive Brain Research* **10**, 145-158 (2000).

**17.** Egner T, Hirsch J. Cognitive control mechanisms resolve conflict through cortical amplification of task-relevant information. *Nature Neuroscience* **8**, 1784-1790 (2005).

**18.** Polk TA, Drake RM, Jonides JJ, Smith MR, Smith EE. Attention enhances the neural processing of relevant features and suppresses the processing of irrelevant features in humans: A functional magnetic resonance imaging study of the stroop task. *Journal of Neuroscience* **28**, 13786-13792 (2008).

**19.** Decety J, Grèzes J. Neural mechanisms subserving the perception of human actions. *Trends in cognitive sciences* **3**, 172-178 (1999).

**20.** Goodale MA, Milner AD. Separate visual pathways for perception and action. *Trends in Neurosciences* **15**, 20-25 (1992).

**21.** Gallese V, Fogassi L, Fadiga L, Rizzolatti G. Action representation and the inferior parietal lobule In: Prinz W, Hommel B, eds. *Attention and performance XIX*. Oxford Oxford University Press; 2002:247-266.

**22.** Shen Y, Xue S, Wang K, Qiu J. Neural time course of emotional conflict control: An ERP study. *Neuroscience letters* **541**, 34-38 (2013).
